# Testing the sexual imagination hypothesis for gender differences in response to infidelity

**DOI:** 10.1186/1756-0500-7-860

**Published:** 2014-11-29

**Authors:** Tsukasa Kato

**Affiliations:** Department of Social Psychology, Toyo University, 5-28-20 Hakusan Bunkyo-ku, Tokyo, 112-8606 Japan

**Keywords:** Sexual infidelity, Jealousy, Evolutionary psychology, Sexual imagination hypothesis, Gender difference

## Abstract

**Background:**

Evolutionary psychologists hypothesized that men are more upset by sexual infidelity than women are, whereas women are more upset by emotional infidelity than men are. On the other hand, the sexual imagination hypothesis states that gender differences in infidelity responses are derived from explicit men’s sexual imagery. Based on the latter hypothesis, we hypothesized that although men would report being more distressed by sexual infidelity than women who were not in a committed relationship (NCR), no gender difference would be reported in a committed relationship (CR).

**Findings:**

These two hypotheses were tested with 598 participants in a CR and 1,643 participants in a NCR. No significant gender difference was found sexual infidelity response in the CR group (*d* = 0.008, a power of .956), whereas men were more upset than women about sexual infidelity in the NCR group. Moreover, a significant interaction between gender and infidelity type was found in the NCR, whereas no significant interaction between gender and infidelity type was observed in the CR group (partial *η*^2^ = 0.005, a power of .943).

**Conclusions:**

Our findings supported the sexual imagination hypothesis but were inconsistent with the EJM hypothesis.

## Findings

The sexual imagination hypothesis regarding gender differences in infidelity responses was tested by comparing individuals who were in a committed relationship (CR) with individuals who were not in a committed relationship (NCR). Our results supported the sexual imagination hypothesis.

## Background

According to some evolutionary psychologists [1–3], men are more upset by a mate’s sexual infidelity than women are, whereas women are more upset by a mate’s emotional infidelity than men are. In the ancestral history of humans, such sex differences are ascribed to the need for reproductive fitness. Ancestral males could not be certain that a putative offspring was their own. For males, a mate’s sexual infidelity poses the risk of investing resources unknowingly in the offspring of rivals. Therefore, males are more vigilant about their mate’s sexual infidelity to prevent this. However, ancestral females incur no such risk because they do not doubt that the putative offspring is their own. However, females face a different risk, that is, emotional infidelity, wherein their mates invest resources in the offspring of their rivals. This evolutionary view is referred to as the sex-specific evolved jealousy mechanism (EJM). Based on the EJM hypothesis, Buss et al. [1] asked survey participants to imagine their partner’s infidelity using brief scenarios and then identify the more distressing of the two types of infidelity. Gender differences in responses to a partner’s infidelity were reported; men were more upset by sexual infidelity than women were, and women were more upset by emotional infidelity than men were. Such differences in infidelity responses have been found repeatedly (for reviews, see [4, 5]).

However, some researchers have questioned the validity of the EJM hypothesis due to contradictory findings [6–10] and alternative interpretations to account for the gender differences in infidelity response. The sexual imagination hypothesis [10, 11] states that gender differences in infidelity responses are derived from men’s explicit sexual imagery [10, 11]. According to this hypothesis, men and women will not differ in responses to a partner’s sexual infidelity, if women too can imagine sexual infidelity as vividly and realistically as men. However, men are more upset by a partner’s sexual infidelity than women are, because men are more likely than women to imagine explicit details (for a review, see [10]). For example, Geer and McGlone [12] found that men were faster and more accurate in recognition tasks involving erotic sentences than women are. Schützwohl and Koch [13] showed that men recalled more cues to sexual infidelity than women did. In addition, data from men and women who have actually experienced infidelity showed no significant gender differences in sexual infidelity reactions [14–18]. Moreover, Kato [10] found that there were no significant gender differences in jealousy when sexual infidelity was imagined in a laboratory using vivid infidelity scenarios and photographs to induce detailed explicit imagery of a partner’s infidelity.

In the present study, gender differences in response to a partner’s sexual infidelity were tested in two groups: those who were currently in a committed relationship (CR) and those who were not in a committed relationship (NCR). Based on the sexual imagination hypothesis, we hypothesized that although men in a NCR group would report being more distressed than women because men are better able to imagine sexual infidelity than women, no gender difference would be reported in a CR group. Previous studies [7, 19–21] have reported that relationship status was a predictor of gender differences in jealousy. Some [7, 19] of these studies found that women in a CR were more distressed or upset over sexual infidelity than women in a NCR. Some studies [7, 19, 21] showed, among married individuals or those in a CR, no significant gender differences in sexual infidelity reactions. In addition, previous studies [19] using Buss’s [1] infidelity scenarios showed that individuals in a NCR reported more difficulty imagining aspects of infidelity than those in a CR. For example, our analysis of Becker et al.’s [19] data revealed that the difference between individuals in a CR and those in a NCR in imagining infidelity was large effect size (Cohen’s *d* = 1.14). However, previous studies that showed no significant gender differences in jealousy about sexual infidelity did not take into account the Type II or beta (*β*) error probability of falsely retaining an incorrect null hypothesis with relatively small sample sizes (about from 200 to 350). We also tested the EJM hypothesis using responses to emotional infidelity as well as sexual infidelity in CR and NCR groups.

## Methods

### Participants and procedure

Participants were 2,241 college students (1,449 women and 792 men; *M* = 19.48 years, SD = 1.52) enrolled in introductory psychology classes. All participants were born in Japan and identified their ethnicity as Japanese. Participants who were currently in a committed relationship were 404 women and 194 men (*M* = 19.80 years, SD = 1.63); in the present study, we refer to this group as the CR group. Participants who were not in a committed relationship were 1,045 women and 598 men (*M* = 19.37 years, SD = 1.47); we refer to this group as the NCR group. Twenty-one potential participants did not provide their gender or relationship status; therefore, they were not included in the study. No one in either group had been married. A committed relationship in the present study referred to a serious and potentially long-lasting romantic committed heterosexual relationship, which did not include casual dating. After giving informed consent, they completed a set of questionnaires in small groups supervised by research assistants. They received course credit for their participation. All procedures were in accordance with the ethical standards of the responsible committee on human experimentation (institutional and national) and with the Helsinki Declaration of 1975, as revised in 2000; this project was approved by the Institutional Ethics Committee of Toyo University.

### Measures

Participants were asked questions about their reactions to infidelity scenarios by Buss et al. [1]. The scenarios were as follows: *Please think of a serious committed romantic relationship that you have had in the past*, *that you currently have*, *or that you would like to have. Imagine your partner enjoying passionate sexual intercourse with that other person. Imagine your partner forming a deep emotional attachment to that person*. Participants were instructed to rate how upset or distressed they would be by each type of infidelity on a 6-point Likert-type scale, ranging from 1 (not at all upset or distressed) to 6 (extremely upset or distressed). Jealousy was assessed using continuous measures for two reasons. First, evolutionary psychologists have employed such scales to demonstrate sex differences in jealousy (for reviews see [5, 9]). Second, there are serious methodological issues with hypothetical forced-choice findings [9]; for example, they are incapable of independently assessing sexual and emotional jealousy. We assessed sexual infidelity independently in order to test the sexual imagination hypothesis. The two infidelity scenarios were presented in random order. No significant effects of presentation order for sexual infidelity (*t*(2239) = 0.16, *p* = .88) and emotional infidelity (*t*(2239) = 0.81, *p* = .42) scores were found.

These infidelity scenarios were originally written in English and translated into Japanese using the back translation method by Kato [10]. Using this Japanese version revealed a similar pattern to Buss et al.’s main findings [1], Kato [10] suggested that his Japanese version was a credible measure to use.

### Data analysis

A 2 (gender) × 2 (group: CR vs. NCR groups) × 2 (infidelity type) analysis of variance (ANOVA) was conducted to test the sexual imagination hypothesis and the EJM hypothesis. In order to test the former hypothesis, planned comparisons for an interaction between gender and group on sexual infidelity response scores were conducted. In addition, planned comparisons for an interaction between gender and infidelity type were conducted for each infidelity type to test the latter hypothesis.

According to Sagarin [22], evidence of an interaction between gender and type of infidelity would demonstrate the EJM hypothesis when participants used continuous rating scales to estimate their distress over the two types of infidelity, which need not show a cross-over pattern (see [9]). However, Harris [9] stated that slopes for men and women should be in opposite directions or show a cross-over interaction in order to document the EJM hypothesis. Sagarin supported the interpretation of EJM-based gender differences in jealousy, whereas Harris is skeptical about the EJM hypothesis. We tested the evolutionary explanation for gender differences using Sagarin’s [22] approach; that is, we examined interactions between gender and infidelity type in the CR and NCR groups. If the EJM hypothesis, both groups should show a significant interaction between gender and infidelity type at minimum.

## Results

Means and standard deviations of responses to sexual and emotional infidelity are shown in Table 1. The 2 × 2 × 2 ANOVA revealed a significant three-way interaction at *p* < .05, *F*(1, 2237) = 10.12, partial *η*^2^ = 0.005.

**Table 1:** **Means and standard deviations of responses to sexual and emotional infidelity**

		Men			Women	
Group	*N*	*M*	*SD*	*N*	*M*	*SD*
Sexual infidelity						
CR	194	4.68	1.31	404	4.67	1.20
NCR	598	4.64	1.17	1045	4.40	1.03
Emotional infidelity						
CR	194	4.63	1.20	404	4.79	1.04
NCR	598	4.40	1.10	1045	4.68	0.92

*Note*. CR = group who currently in a committed relationship. NCR = group who not in a committed relationship.

### Sexual imagination hypothesis

**Figure 1:**
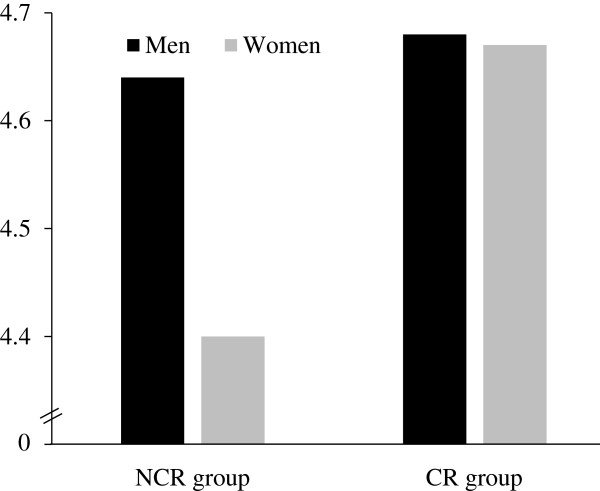
**Means of Responses to sexual infidelity in the group who currently in a committed relationship (CR) and the group who not in a committed relationship (NCR).**

A significant interaction between gender and group with sexual infidelity responses was found at *p* < .05: *F*(1, 2237) = 4.11, partial *η*^2^ = 0.002; but no significant interaction with emotional infidelity responses was found at *p* < .05 (see Figure 1): *F*(1, 2237) = 1.39, partial *η*^2^ = 0.001. Planned comparisons for the interaction revealed that women in the CR group (*M* = 4.67, SD = 1.20) reported being more upset or distressed than women in the NCR group (*M* = 4.40, SD = 1.03), 95% confidence interval (CI) [0.152, 0.400] for the difference scores, *d* = 0.27. Men in the NCR group (*M* = 4.64, SD = 1.17) reported being more upset or distressed than women in the NCR group (*M* = 4.40, SD = 1.03), 95% CI [0.129, 0.347], *d* = 0.24, but there was no such significant gender difference in the CR group (*p* = .947, 95% CI [-0.205, 0.219], *d* = 0.008). In addition, a post-hoc power analysis for the gender differences in the CR using the G*Power program version 3.1.7 [23] revealed a power of .956, based on an effect size of Cohen’s *d* = 0.008 and a total sample size of *N* = 598. The 95% CI [-0.205, 0.219] for the difference scores was narrow. These results were consistent with our expectations.

### EJM hypothesis

A significant interaction between gender and infidelity type in the NCR group was found (*F*(1, 1641) = 87.34, partial *η*^2^ = 0.051), but no significant interaction in the CR group was found at *p* < .05 (*F*(1, 596) = 2.79, partial *η*^2^ = 0.005). The NCR group result was consistent with the EJM hypothesis, but the CR group result was inconsistent with the EJM hypothesis. In addition, a post-hoc power analysis for the interaction in the CR group revealed a power of .943, based on an effect size of partial *η*^2^ = 0.005 and a total sample size of *N* = 2,241.

## Discussion

Planned comparisons for the interaction between gender and group revealed that men reported being significantly more upset or distressed in sexual infidelity responses than did women in the NCR group, whereas there was no significant gender difference in the CR group and a small effect size (Cohen’s *d* = 0.008) and narrow 95% CI for the difference scores. The power analysis for the gender difference in the CR group showed a high value (power = .956), indicating the high probability that its null hypothesis will be rejected given that it is in fact false. That is, the type II error probability is low. Our findings were consistent with previous studies [7, 19, 21] and supported the validity of the sexual imagination hypothesis.

In addition, the interaction between gender and infidelity type in the NCR group was significant, whereas it was not significant in the CR group with a small effect size (partial *η*^2^ = 0.005). The CR group result was inconsistent with the evolutionary prediction for gender differences in jealousy. The power (1 - β) was .943 for the CR group interaction, indicating that the type II error probability is low. If the evolutionary explanation is valid, gender differences in jealousy should be observed among individuals in a CR rather than those in a NCR, because the evolutionary interpretation explains gender differences in jealousy for human couples but not for noncoupled males and females. The results in our sample did not support the EJM hypothesis. Although our data cannot categorically deny the possibility of the evolutionary explanation for gender differences in responses to a partner’s infidelity, our findings indicate that the EJM hypothesis cannot provide an explanation for gender differences in infidelity in our sample.

## Conclusions

We found no significant gender differences in response to a partner’s sexual infidelity the CR group. In addition, no significant interaction between gender and infidelity type was found in the CR group. Our findings supported the sexual imagination hypothesis, but were inconsistent with the EJM hypothesis.

## Abbreviations

CR: individuals who were in a committed relationship
EMJ: sex-specific evolved jealousy mechanism
NCR: individuals who were not in a committed relationship.
